# Measuring the quality of care in nursing home residents with early-onset neurodegenerative diseases: a scoping review

**DOI:** 10.1186/s12904-020-0528-0

**Published:** 2020-02-27

**Authors:** Joyce C. F. Heffels, Irma H. J. Everink, Mayke Oosterloo, Raymund A. C. Roos, Jos M. G. A. Schols

**Affiliations:** 1 Department of Health Services Research and Care and Public Health Research Institute (CAPHRI), Maastricht University, Maastricht, The Netherlands; 2 Huntington Disease Center Land van Horne, Vogelsbleek 1, 6001 BE Weert, The Netherlands; 3 Department of Neurology, Maastricht University Medical Center, Maastricht, The Netherlands; 4 Department of Neurology, Leiden University Medical Center, Leiden, The Netherlands; 5 Department of Family Medicine and Care and Public Health Research Institute (CAPHRI), Maastricht University, Maastricht, The Netherlands

**Keywords:** Early-onset neurodegenerative diseases, Quality of care, Resident, Informal caregiver, Formal caregiver, Perspective, Nursing home, Measurement

## Abstract

**Background:**

Nursing home residents with early-onset neurodegenerative diseases are often younger in comparison with other residents, and need different, often more complex care. Accordingly, the measurements currently used for measuring quality of care in nursing homes may not be suitable for use in this target group. Little is known about the experiences of these residents and of their (in) formal caregivers regarding the quality of care they receive. Therefore, the aim of this scoping review is to explore which instruments are available for measuring the quality of care for nursing home residents with early-onset neurodegenerative diseases (excluding dementia), from the perspective of the resident and of (in) formal caregivers.

**Methods:**

A literature search was performed in the databases Pubmed, Embase, Web of Science and Cinahl. The search strategy consisted of four main concepts: neurodegenerative diseases, quality of care, nursing homes and perspectives of residents, (in) formal caregivers. Studies were included if they used instruments and/or strategies to measure quality of care, focused on nursing home residents with early-onset neurodegenerative diseases and the perspective of either the resident or (in) formal caregiver.

**Results:**

From a total of 809 identified articles, 87 full text articles were screened for eligibility. Five studies were included, only one of which described an instrument. The other four used topic lists and/or themes to measure quality of care. In total, 60 items related to quality of care could be derived. From these 60 items, eight overarching domains were found, with a subdivision into items derived, respectively, from the residents’, informal and formal caregivers’ perspective: ‘emotional support’, ‘physical support’, ‘social support’, ‘care’, ‘care content’, ‘expertise’, ‘communication’ and ‘organization of care’.

**Conclusions:**

Currently, there are no methods for assessing the quality of care specifically focused on nursing home residents with early-onset neurodegenerative diseases. Therefore, the items retrieved in this review give an overview of important topics for measuring the quality of care for this target group, from the perspective of the resident, and of the informal and formal caregivers. These items might be used to develop a tailored instrument for assessing the quality of care for nursing home residents with early-onset neurodegenerative diseases.

## Background

Patients suffering from early-onset neurodegenerative diseases other than pure dementia, such as Parkinson’s Disease (PD) [1], Multiple Sclerosis (MS) [2], Motor Neuron Disease (MND) (e.g. Amyotrophic Lateral Sclerosis) [3] or Huntington’s Disease (HD) [4], show progressive neurological dysfunction. As no cure is available, only symptomatic treatment is possible, which leads to a large burden of care. Most patients need complex care as their disease progresses. When home care is insufficient and no longer feasible, admission to a nursing home is often unavoidable. Several studies show that a substantial part of patients with PD, HD, MS and MND finally reside in a nursing home [1, 2, 5–7].

Patients suffering from neurodegenerative diseases (in this study we refer to neurodegenerative diseases other than pure dementia) living in a nursing home often have different needs than ‘regular’ nursing home residents. Most patients with neurodegenerative diseases have an early onset and therefore a prolonged trajectory of the disease. As a consequence, the majority of these patients is admitted to a nursing home at a relative young age in comparison with the average, much older nursing home resident. Usually they are in a different stage of life; have a partner and growing children. In comparison with isolated dementias, such as Alzheimer’s disease, the neurodegenerative diseases at stake require more specific activities, with regard to the physical, psychological, social and environmental support, mainly because of the early onset and its related impact on daily life and prognosis.

In the Netherlands, there are specialized care units in nursing homes for patients with neurodegenerative diseases such as HD. These specialized care units are more equipped to deliver patient-centered care with regard to knowledge, staff experience and physical environment. However, not much is known about the actual quality of care (QoC) delivered in these units. Although a few studies describe characteristics and factors that contribute to the institutionalization of residents with specific neurodegenerative diseases [2, 4, 5, 8] and their quality of life, little is known about the actual experiences of these residents regarding the QoC they receive in such nursing homes.

To assess the QoC in such specialized care units for residents with neurodegenerative diseases, it is important to gain more insight into residents’ experiences. Furthermore, it is noted in previous literature that informal caregivers (e.g. family members) and formal caregivers (professionals) experience a major care burden in providing care for these residents [9–11]. Accordingly, it is also important to investigate the personal experiences of informal and formal caregivers regarding QoC.

Measuring QoC in nursing homes is often done using instruments such as the Consumer Quality Index (CQI) or other quantitative quality indicators [12]. However, it is debatable whether or not such instruments acknowledge the specific issues that residents with neurodegenerative diseases face in daily life. Such quantitative instruments may not be suitable for assessing the quality of long-term care [13].

The aim of this scoping review is to look at the instruments available for measuring the QoC for nursing home residents with early-onset neurodegenerative diseases from the perspective of the resident and of both formal and informal caregivers. In this way, we hope to provide an answer as to which method would be best to assess the personal experience of residents in nursing homes regarding the care they receive.

## Methods

### Study design

This study is a scoping review, which focuses on gaining as much information as possible about the key concepts of a topic. The framework proposed by the Joanna Briggs Institute [14] for performing scoping reviews, described below, was followed.

### Search strategy

In October 2018, a search was performed in four databases (Pubmed, Embase, Web of Science and Cinahl). The search strategy used consisted of four main concepts: the selected neurodegenerative diseases, quality of care, nursing homes and perspectives of patients, informal caregivers and nursing home staff. In line with this, all possible MeSH terms and synonymous terms where composed. The full search strategy can be found in an additional file (see Additional file 1).

### Study selection

To be included in the review, studies had to meet the following inclusion criteria:

- Studies describing instruments and/or strategies for measuring QoC*;*
- Studies with an outcome measurement and/or strategies for measuring QoC;
- Studies with a focus on residents with early-onset neurodegenerative diseases (PD, MS, MND, HD);
- Studies including nursing home residents or institutional long-term care residents;
- Studies focusing on the perspective of either the resident or of the informal or formal caregiver;
- Studies published between October 2008 and October 2018.

Since developments related to QoC changed rapidly during the last decade, in favor of the patient’s perspective, articles before October 2008 were excluded. In addition, case reports, conference abstracts, as well as studies written in a language other than English or Dutch were excluded. Furthermore, studies focusing on isolated dementia were excluded, as we were specifically interested in other target groups in the nursing home setting with early-onset neurodegenerative disorders with cognitive as well as physical deterioration. In addition, pure dementia usually has a much later onset in life and only shows physical disability in the end stage of the disease.

All articles retrieved from the literature search were imported into Endnote. The first author (JH) screened all titles and abstracts. Furthermore, an independent assessment of a sample of all abstracts (10%) was done by IE, resulting in an expansion of another 10% of all abstracts until an interrater agreement of 95% was reached. Studies without an available abstract were screened on title only in the first round. All abstracts and titles for which no initial consensus on inclusion or exclusion was reached by both authors were included in the full-text round.

In the second round JH screened all available full text articles. If the full text of an article was not available online or in the Maastricht University library, the authors of the study were contacted by email. Again, an independent assessment of 10% of consecutive samples of the included studies was done by IE until a 95% interrater agreement between JH and IE was reached. In case both authors failed to reach consensus regarding inclusion, those articles were discussed with JS. Reasons for exclusion were recorded. Finally, the reference lists of included articles were screened for additional studies to ensure that no relevant publications were missed.

### Methodological quality assessment of included articles

The methodological quality of all included studies was evaluated using the quality assessment criteria developed by Kmet et al. [15]. This instrument included separate scoring systems for the quality of qualitative and for quantitative research designs. For quantitative studies, 14 items covering domains such as appropriate design and sample size were evaluated and scores were allocated. Qualitative studies were scored on 10 items such as appropriate study design and credibility of the study. Both scoring systems provide a range in scoring from 0 to 1.00 as the maximum achievable score [15].

The range of scores for both qualitative and quantitative studies were categorized by the authors of this review as being poor (0–0.25), moderate (0.25–0.50), adequate (0.50–0.75) or outstanding (0.75–1.00). The complete list of both the quantitative and qualitative assessment can be found in additional files (see Additional file 2 and Additional file 3). Two authors (JH and IE) independently assessed the methodological quality of the included studies and discussed differences in scoring until consensus was reached.

### Data extraction

Data were extracted (JH) from the included studies, using a structured data extraction form as seen in Table 1. First, the characteristics of the included studies were extracted (author, year, research topic, target group, settings, methods of data collection and participants). Furthermore, two outcome measures were retrieved from the different studies: a) all items used to assess QoC among nursing home residents with neurodegenerative diseases and their formal and/or informal caregivers, and b) all items assessed by residents and/or (in) formal caregivers of nursing home residents with neurodegenerative diseases as being an important aspect of QoC. All items retrieved were coded (JH) which caused overarching domains to emerge, resulting in further refinement of the data extraction as seen in scoping reviews [14].

**Table 1:** Characteristics of identified studies on measurement of Quality of Care

Author	Year	Research topic	Target group	Settings	Methods of data collection and participants			
					Focus groups	Interviews	Surveys	Other methods
Wilson et al.	2011	Discussions with health care staff about providing palliative and end-of-life care.	MS, PD, MND, HD	Neurological care centers, Hospice	Health care staff (*n* = 77)	Health care staff (*n* = 3)		
Van Rumund et al.	2014	Quality of PD care in Dutch nursing homes from the perspectives of residents, informal caregivers, and professionals.	PD	Nursing homes	Health care staff (*n* = 35)	Residents (*n* = 15), Informal caregivers (*n* = 15)		
Sandsdalen et al.	2016	Patients’ perceptions on the quality of palliative care in and across different care settings.	Among others MS, ALS, PD	Palliative units in nursing homes			Survey of nursing home residents (*n* = 8)*	
Armitage et al.	2009	Residents/ relatives’ perspective on the quality of care provision.	PD	Care homes		Relatives (*n* = 51), Patients (*n* = 24)		
Dellefield et al.	2011	Nursing and interdisciplinary lessons learned in providing care.	HD	Skilled nursing facility				Description of care provision (*n* = 53)

Extraction of the results. *MS* Multiple Sclerosis, *PD* Parkinson’s Disease, *MND* Motor Neuron Disease, *HD* Huntington’s Disease, *ALS* Amyotrophic Lateral Sclerosis. * Maximum of 8 patients, exact number not specified further

## Results

**Fig. 1:**
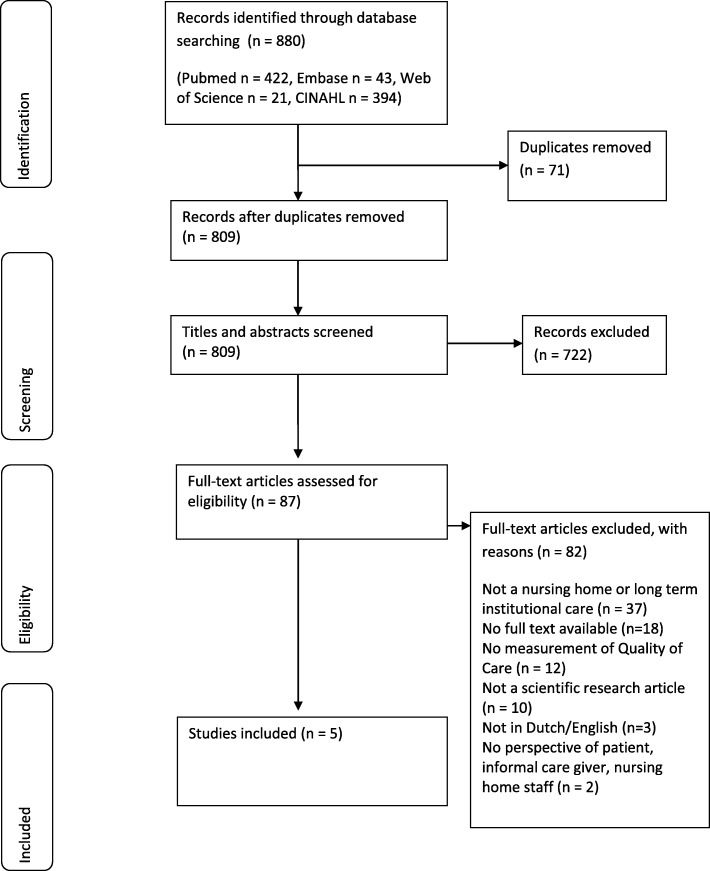
Flowchart of the record identification and selection process

The flowchart (Fig. 1) gives an overview of the study identification process. The search in all databases yielded 809 unique articles. After title and abstract screening, 722 articles were excluded. In total, 87 full text articles were screened for eligibility. The main reasons for exclusion were ‘not nursing home or long-term institutional care’ (*n* = 37), ‘no full text available’ (*n* = 18) and ‘no measurement of quality of care’ (*n* = 12). All other reasons for exclusion (*n* = 15) were described in the flowchart. Finally, five studies were included in this scoping review.

### Characteristics of the articles

The final five articles included in this scoping review were published in 2009 (*n* = 1), 2011 (*n* = 2), 2014 (*n* = 1) and 2016 (*n* = 1). Two of these studies [16, 17] originated in the United Kingdom, one [18] in the USA, one [19] in Norway, and one [1] in the Netherlands. There were four studies [1, 16–18] with a qualitative study design and one study [19] with a quantitative study design. Of the five included studies, one study [18] focused only on HD residents (*n* = 1) and two studies [1, 17] only on PD residents (*n* = 2). One study [19] combined PD residents with MS, MND/ALS residents, and one study [16] combined PD residents with MS, MND/ALS and/or HD residents.

### Methodological quality

Appendix 2 and 3 show the methodological quality of the five included articles. Methodological quality scores ranged from 0.25 to 0.95. One qualitative article was assessed as having ‘poor’ methodological quality [18], one qualitative article as having a ‘moderate’ methodological quality [16] and three articles (two qualitative and one quantitative) as ‘outstanding’ methodological quality [1, 17, 19].

### Data collection in studies

Van Rumund et al. [1] and Wilson et al. [16] both used focus groups of formal caregivers as a method for collecting data. They looked at QoC for residents in nursing homes and hospice/neurological care centers from the perspective of the formal caregiver. In addition, Wilson et al. [16] used interviews with formal caregivers of residents living in a hospice/neurological care center. Van Rumund et al. [1] and Armitage et al. [17] used interviews with residents living in a nursing home and informal caregivers to collect data about QoC. Sandsdalen et al. [19] employed a survey among residents living in a palliative unit of a nursing home to measure QoC from the perspective of residents. In the study of Dellefield et al. [18] a description of the care provision for residents living in a skilled nursing facility was made from the perspective of formal caregivers.

The characteristics of identified studies are presented in Table 1.

### Instruments used in studies

Sandsdalen et al. [19] used the ‘Quality from the patients’ perspective instrument specific to palliative care’ (QPP-PC). The QPP-PC is a 52-item instrument that is divided into four dimensions of quality: ‘medical-technical competence of the caregiver’, ‘physical-technical conditions of the care organization’, ‘identity-orientation approach’ and ‘sociocultural atmosphere’ and three single items about medical care, personal hygiene and atmosphere [19]. The other four studies did not use a specific instrument to assess quality of care, but used topic lists and/or themes to measure QoC [1, 16–18].

### Process and outcome items

Table 2 shows two different results. The first results (the items with a “#”-sign) are the items used in the data collection of the different studies to assess QoC among nursing home residents with neurodegenerative diseases and/or among formal and informal caregivers (process items). The second results (the items with a “+”-sign) are outcome items; these items are the results in the different studies of what residents with neurodegenerative diseases and/or their formal and informal caregivers mention as being important aspects of QoC.

**Table 2:** Data extraction of the identified studies

Domains used to measure quality of care	Items used to measure quality of care	Target group where item is assessed		
		Patients	Informal caregivers	Formal caregivers
Emotional support	Getting to know patient is essential to understanding their wishes and needs [16]			+
	Emotional support [1]	#	#	#
	Emotional support and empathy [1]	+	+	+
	Respect and empathy [19]	#+		
	Honesty [19]	#++		
	Meaningfulness [19]	#		
	Identity-orientation approach [19]	#		
	Spiritual and existential [19]	# (++ lowest score)		
	Emotional wellbeing [17]	#	#	
	Enjoyment, entertainment and well-being [18]			#
	Spirituality [18]			#
	Promotion of dignity [18]			#
	Promotion of autonomy [18]			#
Physical support	Pleasant and safe atmosphere [19]	#++		
	Safety and order [18]			#
Social support	Difficulties with access to community services and equipment [16]			+
	Sociocultural atmosphere [19]	#		
	Relatives and friends [19]	#++		
	Social functioning [17]	#	#	
	Nature of relatives’ involvement [17]	+	+	
	Care home environment and culture [17]	+	+	
	Meaningful social interaction [18]			#
Care	Nursing [1]			#
	Medical treatment [1]			#
	Multidisciplinary care [1]			#
	Treatment [1]	#	#	
	Care [1]	#	#	
	Medical care [19]	#+		
	Participation [19]	# (++ lowest score)		
	Continuity [19]	# (+ lowest score)		
	Care provision [17]	+	+	
Care content	Personal hygiene [19]	#++		
	Access to help, food and equipment [19]	#+		
	Symptom relief [19]	#		
	Exhaustion [19]	# (++ lowest score)		
	Physical functioning [17]	#	#	
	Cognitive functioning [17]	#	#	
	Functional variation [17]	+	+	
	Nutrition [18]			#
	Functional competence [18]			#
	Comfort [18]			#
Expertise	Highlighted importance of teamwork and shared expertise [16]			+
	Not always possible to identify the dying phase [16]			+
	Expertise [1]			#
	Clustering of PD patients [1]	#	#	#
	Staff knowledge on PD-related issues [1]	+	+	+
	Neurologist involvement [1]	+	+	+
	Specialized PD nurse [1]	+	+	+
	Clustering of residents into specialized PD units [1]	+	+	+
	Medical technical competence of caregiver [19]	#		
	Lack of PD information [17]	+	+	
Communication	Complexity of the conditions prove a challenge for care, particularly in terms of communication [16]			+
	Information [19]	# (+ lowest score)		
	Communication [18]			#
Organization of care	Elements of a good death include limiting transfers to hospital [16]			+
	Suggestions for improvement [1]	#	#	#
	Budget and staff occupation [1]	+	+	+
	Physical technical conditions of the care organization [19]	#		
	Planning and cooperation [19]	#		
	Care management [17]	#	#	

#Item in instrument or study used to assess quality of care (process item), +Item assessed by patients, informal caregivers or formal caregivers as being an important concept in measuring quality of care (outcome item)

We found 60 different items, of which 36 were process items and 17 were outcome items. Seven items were used both as process and outcome item and were thus overlapping; these were derived from the same study [19].

### Overarching domains

After coding the 60 different items derived from the included articles, eight overarching domains emerged. These eight domains are: ‘emotional support’, ‘physical support’, ‘social support’, ‘care’, ‘care content’, ‘expertise’, ‘communication’ and, finally, ‘organization of care’. The overarching domains and the different items belonging to these domains are described in Table 2.

## Discussion

This review presents the available research regarding assessment of the QoC of nursing home residents with early-onset neurodegenerative diseases from the perspective of residents and their formal and informal caregivers. The 60 items that were found in the five included studies are merged into eight overarching domains: ‘emotional support’, ‘physical support’, ‘social support’, ‘care’, ‘care content’, ‘expertise’, ‘communication’ and ‘organization of care’.

When comparing our results with research in the field, we found that Sion et al. [20] had developed a framework for conceptualizing the experienced quality of long-term care for older people from the perception of care recipients (INDEXQUAL). This framework evaluates the journey of a person and focuses on their expectations, experiences and assessment of QoC [20]. The INDEXQUAL emphasizes not only the physical, but also the social and emotional aspects of care. This is similar to the domains that are composed in our review, where ‘emotional’ and ‘social support’ appeared to be important aspects for assessing quality of care, along with the domain ‘physical support’. The division of experiences related, respectively, to the care receiver, the professional caregiver and the informal caregiver, within the phenomenon of relationship-centered care, also fit in the three perspectives used in our review. This shows that the approach of our review coincides with current trends in nursing home care.

A study of Borreani et al. [21] focused on needs, views and experiences, perceived by adults with severe MS living at home, their carers and health professionals. The quality of care items found in this study [21] are highly similar to the items found in our review. However, the setting of the study is community care, whereas the setting of our study is institutional long term care. Also, the instruments described in this review do not include the domain ‘health and social policies’ (with categories ‘rights’, ‘culture’ and ‘patient organizations’) found in the study of Borreani et al. [21]. Whereas, the domain ‘expertise’ included in our review was not described in the study of Borreani [21].

Furthermore, Peters et al. [22] investigated patients’ experiences of care services for long-term neurological conditions (LTNC) in MND, MS and PD patients (*n* = 2563) living at home. The questionnaire consisted of six dimensions, divided into 35 items, and was based on relevant quality requirements, guidelines, scientific articles and expert opinions [22]. The majority of the dimensions in this study correspond with the eight domains found in this review. However, the dimensions ‘diagnosis’ and ‘general practitioner’, in the instrument of Peters et al. [22] are not present in the domains of our review. One explanation for this may be the studies are carried out in different settings. Furthermore, the involvement of the general practitioner in nursing homes differs among countries. Finally, the content of the dimension ‘personal care and support’ in the study of Peters et al. [22] is more focused on ‘obtaining financial support and help with housework’ whereas in this study the focus is more on ‘nursing’ and ‘medical care’. Early-onset neurodegenerative diseases such as PD and HD in an advanced stage require a palliative care approach [23, 24] and therefore it is common for care providers to address aspects of advance care planning or end-of-life planning. However, the domain ‘advance care planning’ or ‘end-of-life planning’ was not explicitly found in this review.

### Strength and limitations

The current scoping review has several strengths. First, a comprehensive search strategy was used, minimizing the chance of missing relevant studies. Furthermore, the study selection, as well as the methodological quality assessment, was done by two authors independently, increasing rigor.

Published research in this topic is scarce and therefore this study adds important information. However, this study also demonstrates the current gap in the knowledge and the need for more research. The quality of the studies included in this review ranged from moderate to outstanding and this makes comparison of the findings difficult. It is recommended to take the differences in methodological quality into account when interpreting the data.

Furthermore, the included studies have small sample sizes; one study had less than eight participants. However, due to the subject of our review, and the number of included studies, the size of the sample is less important than the content of the instrument.

To answer our research question, the search was explicitly limited to instruments developed and/or used to measure QoC in nursing home residents with early-onset neurodegenerative diseases. We are aware of the fact that there might be instruments developed and/or used to measure QoC in other settings or target groups (e.g. younger residents with dementia) which were not included in this review. Examples are Patient Reported Outcome Measures (PROMs) that could in time be an option to use within our target group, possibly in adapted form. These instruments will be taken into account in future development of a QoC assessment tool for our target group.

## Conclusion and implications

This review shows that little has been written about the QoC of nursing home residents with an early-onset neurodegenerative disease. Our study is the first combining the perspectives of residents with early-onset neurodegenerative diseases and their informal and formal caregivers to measure QoC. It also demonstrates the current gap of research in this field and the need for further research.

The main results from this review may be interesting for a wide audience working with residents with neurodegenerative diseases, especially those working in a nursing home setting. Based on the results of this study, we recommend developing and validating a tailored instrument to measure the QoC for nursing home residents with early-onset neurodegenerative diseases from the perspective of residents and their formal and informal caregivers, including the domains found in this review. This instrument can be used in the future to assess the impact of interventions on the QoC of these residents in a more tailored way.

## Supplementary information

**Additional file 1.** Search terms.

**Additional file 2.** Methodological quality assessment of qualitative studies.

**Additional file 3.** Methodological quality assessment of quantitative studies.

## Data Availability

All data generated or analyzed during this study are from published articles.

## Abbreviations

CQI: Consumer Quality Index
HD: Huntington’s Disease
LTNC: Long-term neurological conditions
MND: Motor Neuron Disease
MS: Multiple Sclerosis
PD: Parkinson’s Disease
PROMs: Patient Reported Outcome Measures
QoC: Quality of Care
QPP-PC: Quality from the patients’ perspective instrument specific to palliative care
