# Supplementary material for: Measuring the quality of care in nursing home residents with early-onset neurodegenerative diseases: a scoping review
Source: BMC Palliat Care. 2020 Feb 27;19:25. doi: 10.1186/s12904-020-0528-0 (PMC7047396; doi:10.1186/s12904-020-0528-0)
Supplement: Supplementary file 2 — Additional file 2. Methodological quality assessment of qualitative studies. [file 12904_2020_528_MOESM2_ESM.docx]

**Quality Scoring of Qualitative studies**

|  | **Quality Assessment** | **Wilson et al (2011)** | **Van Rumund et al (2014)** | **Armitage et al (2009)** | **Dellefield et al (2011)** |
| --- | --- | --- | --- | --- | --- |
| **1** | **Question/objective clearly described?** | 1 | 2 | 2 | 2 |
| **2** | **Design evident and appropriate to answer study question?** | 2 | 1 | 2 | 0 |
| **3** | **Context for the study is clear?** | 2 | 2 | 2 | 2 |
| **4** | **Connection to a theoretical framework/wider body of knowledge?** | 0 | 1  . | 2 | 1 |
| **5** | **Sampling strategy described, relevant and justified?** | 1 | 2 | 1 | 0 |
| **6** | **Data collection methods clearly described and systematic?** | 1 | 2 | 2 | 0 |
| **7** | **Data analysis clearly described, complete and systematic?** | 0 | 2 | 2 | 0 |
| **8** | **Use of verification procedure(s) to establish credibility of the study?** | 0 | 2 | 2 | 0 |
| **9** | **Conclusions supported by the results?** | 2 | 2 | 1 | 0 |
| **10** | **Reflexivity of the account?** | 0 | 1 | 1 | 0 |
|  | **Total score** | 9/20 = 0,45 | 17/20 = 0,85 | 17/20 = 0,85 | 5/20 = 0,25 |

*‘Yes’=2, ‘Partial’=1, ‘No’=0 score*
