# Supplementary material for: Measuring the quality of care in nursing home residents with early-onset neurodegenerative diseases: a scoping review
Source: BMC Palliat Care. 2020 Feb 27;19:25. doi: 10.1186/s12904-020-0528-0 (PMC7047396; doi:10.1186/s12904-020-0528-0)
Supplement: Supplementary file 3 — Additional file 3. Methodological quality assessment of quantitative studies. [file 12904_2020_528_MOESM3_ESM.docx]

**Quality Scoring of Quantitative studies**

|  | **Quality Assessment** | **Sandsdalen et al (2016)** |
| --- | --- | --- |
| **1** | **Question/objective sufficiently described?** | 2 |
| **2** | **Design evident and appropriate to answer study question?** | 2 |
| **3** | **Method of subject selection or source of information/input variables is described and appropriate.** | 2 |
| **4** | **Subject characteristics or input variables/information sufficiently described?** | 2 |
| **5** | **If random allocation to treatment group was possible, is it described?** | N/A |
| **6** | **If interventional and blinding of investigators to intervention was possible, is it reported?** | N/A |
| **7** | **If interventional and blinding of subjects to intervention was possible, is it reported?** | N/A |
| **8** | **Outcome and (if applicable) exposure measure(s) well defined and robust to measurement/ misclassification bias? Means of assessment reported?** | 2 |
| **9** | **Sample size appropriate?** | 1 |
| **10** | **Analysis described and appropriate?** | 2 |
| **11** | **Some estimate of variance (e.g. confidence intervals, standard errors) is reported for the main results/outcomes** | 2 |
| **12** | **Controlled for confounding?** | 2 |
| **13** | **Results reported in sufficient detail?** | 2 |
| **14** | **Do the results support the conclusions?** | 2 |
|  | **Total score** | 28 – (3x2) = 22  21/22 = 0,95 |

*‘Yes’=2, ‘Partial’=1, ‘No’=0 score, ‘N/A’=items not applicable to particular study design*
